# Knowledge, attitudes, and practices of chronic disease patients in Bojanala towards COVID-19

**DOI:** 10.4102/safp.v65i1.5763

**Published:** 2023-10-30

**Authors:** Beya Mpinda, Cila Dominique Kabogo, Jean Louis Mata Yoko, John Tumbo

**Affiliations:** 1 Department of Family Medicine, Sefako Magkatho Health Sciences University, Pretoria, South Africa

**Keywords:** COVID-19, chronic diseases, knowledge, attitude, practices, primary health care

## Abstract

**Background:**

COVID-19 is an acute respiratory disease. Its morbidity and mortality in patients with comorbidities have been established. This study evaluated the knowledge, attitude and practices regarding COVID-19 of patients with comorbidities attending primary health care services.

**Methods:**

An analytical cross-sectional study was conducted, with data collected from patients using a self-administered questionnaire. Knowledge, attitude and practice scores were calculated. Descriptive and inferential statistical analyses were used, and the level of significance was set at 5%.

**Results:**

This study enrolled 469 participants aged 18–84 years, and the majority were women. The mean knowledge score was 7.09 ± 1.73 out of 9, the mean attitude score was 2.33 ± 0.86 out of 3, and the mean practices score was 3.79 ± 0.56 out of 4. There was a positive linear relationship between knowledge and attitude scores and between attitude and practices scores; as one score increased, the other also increased significantly.

**Conclusion:**

The level of knowledge was good in general, with optimistic attitudes and good practices by the patients. Those aged 70 years and above need special attention because older persons have poorer knowledge of and practices towards COVID-19, which could lead to higher hospitalisation and mortality rates.

**Contribution:**

This study found that patients with chronic diseases had good COVID-19 knowledge, attitudes and practices, while interventions targeting patients aged 70 years and above are needed to improve their COVID-19 awareness and practices.

## Introduction

COVID-19 is a respiratory disease caused by the novel coronavirus first discovered in Wuhan. The World Health Organization (WHO) declared the official name as COVID-19 in February 2020,^1^ and on 11 March 2020, declared the COVID-19 outbreak a pandemic.^2^ The overall mortality rate varies from 0.7% to 10.8%.^1,3^ with close to 200 million confirmed cases and 4 million deaths worldwide. Elderly patients and people with comorbidities suffer severe disease and have a poor prognosis with COVID-19.^3^

A systematic review of the burden of COVID-19 revealed that it was significantly higher in developing countries than in high-income countries. This difference was attributed to a combination of factors, including elevated transmission rates among middle-aged and older adults, as well as limited access to adequate healthcare.^4^

Studies carried out in Africa showed that poor knowledge of COVID-19 was associated with poor practice of measures able to prevent the disease.^2,3^ Internationally, the relatively high knowledge level and positive attitude justified healthy preventive practices towards COVID-19.^5^

Furthermore, studies on knowledge, attitude and practice (KAP) towards COVID-19 have shown that good KAP constitute the backbone of the fight against the disease.^6,7^

The correlation between non-communicable diseases and COVID-19 disease severity has been widely described.^8,9,10,11,12,13,14,15,16^ The middle-aged, the elderly and patients with chronic disease are susceptible. The awareness level of elderly people and/or people living with chronic diseases in Bojanala towards COVID-19 is not known.

This study therefore aimed to assess the KAP towards COVID-19 infections among patients with comorbidities attending primary health care services in Bojanala health district.

## Research methods and design

### Study design

An analytical cross-sectional study was conducted from March 2021 to October 2021 in Bojanala Health District in the North West province.

### Study setting

The survey was conducted in Bojanala in the North West province of South Africa. The study was carried out in the five subdistricts of Rustenburg, Madibeng, Moses Kotane, Morelete and Ketleng.

### Study population and sampling strategy

The population of the Bojanala health district is estimated at 1 779 141, with an incidence of 3.04 COVID-19 cases per 100 000 (district health information system). Participants were patients with chronic diseases attending primary health care services during the period of study. We used a single population proportion formula to determine the sample size. The assumptions were a 5% margin of error, a 95% confidence interval (CI) and a 50% prevalence for KAP.

A total of 469 respondents were selected for the study. This sample was proportionally divided into five to represent the five subdistricts. The systematic random sampling method was used, with every third client after a random start being selected for the study.

### Data collection

Data were collected from patients with comorbidities using a self-administered questionnaire; where this was not possible, a research assistant (professional nurse) assisted the patients in completing the questionnaire.

The questionnaire was developed by authors using the Risk Communication and Community Engagement (RCCE) tool^17,18^ and articles published on knowledge, attitude, practice and associated factors related to COVID-19 before conducting this study.

The final structured questionnaire was piloted using 12 participants in three clinics to assess the adequacy of its contents, participants’ comprehension and potential difficulties.

The questionnaire consisted of three sections: demographics, chronic medical profile and COVID-19 knowledge-attitude-practice (KAP).

The demographics include age in years, gender, marital status, education level and employment status.

The chronic medical conditions were hypertension, diabetes mellitus, asthma, human immunodeficiency virus (HIV) disease, chronic obstructive pulmonary disease, hypercholesterolaemia, epilepsy and mental health condition. The COVID-19 KAP section consisted of nine questions pertaining to knowledge regarding COVID-19 disease, three questions addressing attitudes towards the disease, and four questions covering patients’ practices towards COVID-19.

The Cronbach’s alpha coefficient of the knowledge questionnaire was 0.73, indicating satisfactory internal consistency.^19^

### Data analysis

The data were captured in Microsoft Excel, and data management and analyses were performed using Stata 17. Mean (standard deviation [s.d.]) was used to report continuous variables, while frequencies and percentages were used to summarise categorical variables. The KAP item responses were summarised using frequencies and percentages. Cronbach’s alpha was used to assess the reliability of the KAP items. Knowledge, attitude and practices scores were calculated for each patient with chronic comorbidities by adding their correct answers. A correct answer was scored 1, while an incorrect answer was scored 0. The knowledge score ranged from 0 to 9, the attitude score ranged from 0 to 3, and the practice score ranged from 0 to 4. The scores were summarised using mean (s.d.) and the Pearson correlation test was used to assess the linear relationship between the KAP scores. The scores were further categorised into binary outcomes using the mean as the mid-point for each score. The knowledge score was categorised into good (≥ 7) and poor (< 7); attitude was categorised into positive (≥ 2) and negative (< 2); and the practice score was categorised into good (≥ 4) and poor (< 4). The results for the categorised scores were summarised as frequencies and percentages. The Pearson Chi-square test assessed associations between the KAP binary variables, sociodemographic variables and comorbidity variables. Adjusted logistic regression was used to determine the factors associated with good knowledge, a positive attitude and good practices. The level of significance was set at 5%.

### Ethical considerations

The research protocol was submitted to Sefako Makgatho University Research Ethics Committee (SMUREC), and ethics approval was granted (reference number: SMUREC/M/307/2020:IR). Permission to conduct the study was given by the Bojanala District Health Services. Study participants gave informed consent before they were enrolled. Their identities were kept confidential, thereby ensuring anonymity and confidentiality.

## Results

In this study, 469 patients with chronic comorbidities (chronic patients) responded to the survey. The mean ± s.d. age of these chronic patients was 47.6 ± 15.2 years, ranging from 18 years to 84 years. Most chronic patients fell into the 30–49 year age group (43.28%, *n* = 203), as shown in Table 1. The majority of the chronic patients were women (*n* = 295, 62.9%) and had attained secondary education (60.77%, *n* = 285). There were more single chronic patients in this study (45.84%, *n* = 215), and a few were smokers (17.06%, *n* = 80).

**TABLE 1:** Sociodemographic characteristics of the chronic patients.

Variable	Categories	Frequency	Percentage
Age (years)	< 30	65	13.86
	30–49	203	43.28
	50–69	162	34.54
	70 and above	39	8.32
Gender	Male	174	37.10
	Female	295	62.90
Employment status	Employed	300	63.97
	Unemployed	169	36.03
Educational level	None	35	7.46
	Primary	106	22.60
	Secondary	285	60.77
	Tertiary	43	9.17
Marital status	Single	215	45.84
	Married	172	36.67
	Divorced	28	5.97
	Widowed	38	8.10
	Cohabitating	16	3.41
Smoking	No	389	82.94
	Yes	80	17.06

All of the participants were chronic patients; 322 (68.66%) had only one comorbidity, while the rest had two to four comorbidities. There were 246 (52.45%) hypertensive patients, 78 (16.63%) with diabetes, 25 (5.33%) with asthma, and 268 (52.88%) were HIV-positive (Table 2).

**TABLE 2:** Comorbidities of the chronic patients.

Variable	Categories	Frequency	Percentage
Hypertension	No	223	47.55
	Yes	246	52.45
Diabetes	No	391	83.37
	Yes	78	16.63
Asthma	No	444	94.57
	Yes	25	5.33
HIV-positive	No	221	47.12
	Yes	248	52.88
Chronic obstructive pulmonary disease	No	459	97.87
	Yes	10	2.13
Hypercholesterolaemia	No	466	99.36
	Yes	3	0.64
Epilepsy	No	456	97.23
	Yes	13	2.77
Mental health condition	No	464	98.51
	Yes	7	1.49

HIV, human immunodeficiency virus.

The knowledge about COVID-19 responses is shown in Table 3. Most of the chronic patients were knowledgeable about COVID-19, because more than 70% of them selected the ‘correct’ responses for the knowledge items, except for the mixed responses linked to the treatment of COVID-19, which 44.56% (*n* = 209) said is not available. The average scale reliability for the knowledge items was 0.7286.

**TABLE 3:** Responses to COVID-19 knowledge items.

Knowledge items	Correct		Incorrect		Don’t know		Alpha
	*n*	%	*n*	%	*n*	%	
The main clinical symptoms of COVID-19 are fever, fatigue, dry cough, sore throat and myalgia.	425	90.62	16	3.41	28	5.97	0.71
Most people with comorbidities develop severe COVID-19.	369	78.46	30	6.40	71	15.14	0.71
Does the COVID-19 virus spread via respiratory droplets and contact?	417	88.91	20	4.26	32	6.82	0.68
Facemasks and social distancing reduce the spread of COVID-19.	447	95.31	13	2.77	9	1.92	0.71
To prevent infection by COVID-19, individuals should avoid going to crowded places such as stadiums and bars.	446	95.10	12	2.56	11	2.35	0.71
There is a treatment for COVID-19.	126	26.87	209	44.56	134	28.57	0.75
Are vaccines against COVID-19 available in South Africa?	418	89.13	24	5.12	27	5.76	0.70
Patients with underlying chronic diseases are at a high risk of infection.	346	73.77	47	10.02	76	16.20	0.67
People with comorbidity are at high risk of death from COVID-19 compared to people without comorbidity.	334	71.22	47	10.02	88	18.76	0.68

The responses regarding COVID-19 practices and attitude are shown in Table 4. Most of the chronic patients indicated good practices about COVID-19, because more than 89% of them selected the ‘correct’ responses for the practice items. However, the average scale reliability for the knowledge items was 0.46. Regarding attitude, most of the chronic patients indicated a positive attitude about COVID-19, because more than 65% of them selected the ‘correct’ responses for the attitude items. However, the average scale reliability for the knowledge items was 0.48.

**TABLE 4:** Response to COVID-19 practice and attitude items.

Practice and attitude items	Yes		No		Not sure		Alpha
	*n*	%	*n*	%	*n*	%	
**Practice items**							
I use soap and water to wash my hands after coughing or sneezing or touching contaminated objects such as tissues	455	97.01	9	1.92	5	1.07	0.36
I avoid touching my face (eyes, nose or mouth) with uncleaned hands	441	94.03	13	2.77	15	3.20	0.28
I use a face mask in the crowds, and I visit healthcare settings and shops	461	98.29	6	1.28	2	0.43	0.40
I will have the COVID-19 vaccine once available	419	89.34	21	4.48	29	6.18	0.53
**Attitude items**							
Do you agree that COVID-19 will finally be successfully controlled?	308	65.67	62	13.22	99	21.11	0.29
If you test positive, will you agree to be isolated from the community?	407	86.78	42	8.96	20	4.26	0.50
If a corona vaccine is available, would you have it?	376	80.17	15	3.20	78	16.63	0.27

In general, the mean ± s.d. knowledge score was 7.09 ± 1.73 and ranged from 0 to 9. The mean ± s.d. attitude score was 2.33 ± 0.86 and ranged from 0 to 3. The mean ± s.d. practice score was 3.79 ± 0.56 and ranged from 0 to 4. Table 5 shows the correlation analysis of KAP scores. There was a significant positive linear relationship between the knowledge and attitude scores (*r* = 0.1725, *p* = 0.0002), knowledge and practice scores (*r* = 0.2986, *p* < 0.0001) and attitude and practice scores (*r* = 0.2591, *p* < 0.0001). As one score increases, the other also increases significantly.

**TABLE 5:** Correlation matrix of knowledge, attitude and practice scores.

KAP items	Knowledge score	Attitude score	Practice score
Knowledge score	1	-	-
Attitude score	0.1725	1	-
*p*	0.0002	-	-
Practice score	0.2986	0.2591	1
*p*	< 0.0001	< 0.0001	-

KAP, knowledge, attitude and practices.

The KAP scores were categorised based on the mean cut-off values. Values ≥ mean were considered to show good knowledge (≥ 7), positive attitude (≥ 2) and good practice (≥ 4). Of the chronic patients, 71% (*n* = 333) had good knowledge about COVID-19, 55.86% (*n* = 262) had a positive attitude towards COVID-19, and 84.43% (*n* = 396) had good practices for COVID-19.

Associations between KAP categorical variables and sociodemographic and comorbidity variables were assessed using the Chi-square test (Table 6). There was a significant association between knowledge about COVID-19 and age group, as chronic patients aged 70 years and above did not have good knowledge (58.97%, *p* < 0.0001); employment status, as those who were unemployed had good knowledge (77.51%, *p* = 0.02); educational level, as those with tertiary education had good knowledge (79.07%, *p* < 0.0001); being hypertensive, as those who were not hypertensive had good knowledge (76.23%, *p* = 0.017); and HIV status, as those who were HIV-positive had good knowledge (78.23%, *p* < 0.0001).

**TABLE 6:** Associations between participants’ characteristics and knowledge, attitude and practices.

Variable	Knowledge					Attitude					Practices				
	Poor		Good		*p*	Negative		Positive		*p*	Poor		Good		*p*
	*n*	%	*n*	%		*n*	%	*n*	%		*n*	%	*n*	%	
**Age (years)**					**< 0.0001**					0.064					**0.012**
< 30	13	20.00	52	80.00	-	30	46.15	35	53.85	-	10	15.38	55	84.62	-
30–49	48	23.65	155	76.35	-	102	50.25	101	49.75	-	20	9.85	183	90.15	-
50–69	52	32.10	110	67.90	-	59	36.42	103	63.58	-	33	20.37	129	79.63	-
70 and above	23	58.97	16	41.03	-	16	41.03	23	58.97	-	10	25.64	29	74.36	-
**Gender**					0.338					0.317					0.302
Male	55	31.61	119	68.39	-	82	47.13	92	52.87	-	31	17.82	143	82.18	-
Female	81	27.46	214	72.54	-	125	42.37	170	57.63	-	42	14.24	253	84.76	-
**Employment status**					**0.02**					0.151					**0.006**
Employed	98	32.67	202	67.33	-	125	41.67	175	58.33	-	57	19.00	243	81.20	-
Unemployed	38	22.49	131	77.51	-	82	48.52	87	51.48	-	16	9.47	153	90.53	-
**Educational level**					**< 0.0001**					0.605					**< 0.0001**
None	23	65.71	12	34.29	-	19	54.29	16	45.71	-	13	37.14	22	62.86	-
Primary	40	37.74	66	62.26	-	48	45.28	58	54.72	-	20	18.87	86	81.13	-
Secondary	64	22.46	221	77.54	-	121	42.46	164	57.54	-	33	11.58	252	88.42	-
Tertiary	9	20.93	34	79.07	-	19	44.19	24	55.81	-	7	16.28	36	83.72	-
**Marital status**					0.114					0.122					0.061
Single	54	25.12	161	74.88	-	88	40.93	127	59.07	-	24	11.16	191	88.84	-
Married	54	31.40	118	68.60	-	77	44.77	95	55.23	-	29	16.86	143	83.14	-
Divorced	6	21.43	22	78.57	-	12	42.86	16	57.14	-	8	28.57	20	71.43	-
Widowed	17	44.74	21	55.26	-	18	47.37	20	52.63	-	9	23.68	29	76.32	-
Cohabitating	5	31.25	11	68.75	-	12	75.00	4	25.00	-	3	18.75	13	81.25	-
**Smoking**					0.746					0.676					0.6
No	114	29.31	275	70.69	-	170	43.70	219	56.30	-	59	15.17	330	84.83	-
Yes	22	27.50	58	72.50	-	37	46.25	43	53.75	-	14	17.50	66	82.50	-
**Hypertension**					**0.017**					**0.002**					0.941
No	53	23.77	170	76.23	-	115	51.57	108	48.43	-	35	15.70	188	84.30	-
Yes	83	33.74	163	66.26	-	92	37.40	154	62.60	-	38	15.45	208	84.55	-
**Diabetes**					0.706					0.886					0.187
No	112	28.64	279	71.36	-	172	43.99	219	56.01	-	57	14.58	334	85.42	-
Yes	24	30.77	54	69.23	-	35	44.87	43	55.13	-	16	20.51	62	79.49	-
**Asthma**					0.571					0.989					0.951
No	130	29.28	314	70.72	-	196	44.14	248	55.86	-	69	15.54	375	84.46	-
Yes	6	24.00	19	76.00	-	11	44.00	14	56.00	-	4	16.00	21	84.00	-
**HIV-positive**					**< 0.0001**					**0.001**					0.153
No	82	37.12	139	62.90	-	80	36.20	141	63.80	-	40	18.10	181	81.90	-
Yes	54	21.77	194	78.23	-	127	51.21	121	48.79	-	33	13.31	215	86.69	-

Note: Bold indicate significance at 5%.

HIV, human immunodeficiency virus.

There was a significant association between a positive attitude towards COVID-19 and being hypertensive, as those who were hypertensive had a positive attitude (62.6%, *p* = 0.002); and HIV status, as those who were HIV-negative had a positive attitude (63.8%, *p* = 0.001). There was a significant association between good practices for COVID-19 and employment status, as those who were unemployed had good practices (90.53%, *p* = 0.006); and educational level, as those with secondary education had good practices (88.42%, *p* < 0.0001).

Table 7 shows the adjusted logistic results to determine factors associated with good knowledge, a positive attitude and good practices. Level of education was the only variable associated with good knowledge in this analysis. The higher the level of education the chronic patients had, the increased odds of good knowledge about COVID-19. Compared to those who had no education, those who had primary education had increased odds of 3.2 (95% CI: 1.39–7.38) of having good knowledge, those who had secondary education had increased odds of 5.05 (95% CI: 2.23–11.4) of having good knowledge and those who had tertiary education had increased odds of 5.06 (95% CI: 1.66–15.45), adjusting for other variables.

**TABLE 7:** Regression results to predict good knowledge, a positive attitude and good practices.

Variable	Knowledge			Attitude			Practice		
	AOR	95% CI	*p*	AOR	95% CI	*p*	AOR	95% CI	*p*
**Age (years)**									
< 30	1 (base)	-	-	1 (base)	-	-	1 (base)	-	-
30–49	0.88	0.41–1.85	0.732	0.94	0.51–1.72	0.832	1.67	0.68–4.11	0.263
50–69	0.82	0.35–1.93	0.647	1.54	0.73–3.24	0.253	0.95	0.34–2.66	0.929
70 and above	0.41	0.13–1.25	0.116	1.46	0.52–4.12	0.48	0.97	0.25–3.75	0.969
**Gender**									
Male	1 (base)	-	-	1 (base)	-	-	1 (base)	-	-
Female	1.23	0.78–1.96	0.375	1.11	0.72–1.67	0.65	1.16	0.66–2.03	0.603
**Employment status**									
Employed	1 (base)	-	-	1 (base)	-	-	1 (base)	-	-
Unemployed	1.15	0.69–1.92	0.590	0.89	0.57–1.39	0.608	1.81	0.91–3.59	0.093
**Educational level**									
None	1 (base)	-	-	1 (base)	-	-	1 (base)	-	-
Primary	3.20	1.39–7.38	**0.006**	1.53	0.69–3.41	0.3	2.31	0.95–5.61	0.064
Secondary	5.05	2.23–11.4	**< 0.001**	2.19	1.00–4.82	**0.049**	3.13	1.29–7.61	**0.011**
Tertiary	5.06	1.66–15.45	**0.004**	2.38	0.87–6.54	0.093	1.89	0.55–6.47	0.311
**Marital status**									
Single	1 (base)	-	-	-	-	-	1 (base)	-	-
Married	1.03	0.61–1.73	0.923	0.57	0.36–0.92	**0.021**	0.63	0.32–1.24	0.18
Divorced	1.74	0.61–5.03	0.307	0.71	0.29–1.69	0.443	0.34	0.12–0.94	**0.039**
Widowed	0.97	0.42–2.27	0.951	0.48	0.21–1.07	**0.073**	0.58	0.21–1.62	0.299
Cohabitating	0.84	0.26–2.77	0.775	0.27	0.08–0.91	**0.034**	0.47	0.11–1.98	0.301
**Smoking**									
No	1 (base)	-	-	1 (base)	-	-	1 (base)	-	-
Yes	1.21	0.66–2.23	0.542	1.06	0.62–1.84	0.824	0.98	0.48–2.02	0.966
**Hypertension**									
No	1 (base)	-	-	1 (base)	-	-	1 (base)	-	-
Yes	1.18	0.64–2.19	0.595	1.49	0.88–2.54	0.141	2.46	1.12–5.38	**0.024**
**Diabetes**									
No	1 (base)	-	-	1 (base)	-	-	1 (base)	-	-
Yes	1.43	0.78–2.63	0.247	0.72	0.42–1.24	0.239	0.82	0.41–1.66	0.582
**Asthma**									
No	1 (base)	-	-	1 (base)	-	-	1 (base)	-	-
Yes	1.56	0.54–4.49	0.406	0.81	0.34–1.96	0.645	1.29	0.39–4.25	0.681
**HIV**									
No	1 (base)	-	-	1 (base)	-	-	1 (base)	-	-
Yes	1.81	0.99–3.28	0.052	0.59	0.35–1.01	0.053	1.47	0.69–3.11	0.315

Note: Bold indicate significance at 5%.

AOR, adjusted odds ratio; CI, confidence interval; HIV, human immunodeficiency virus.

Secondary level of education, being married, widowed and cohabitating were factors associated with a positive attitude towards COVID-19. Adjusting for other variables, those who had secondary education had increased odds of having a positive attitude of 2.19 (95% CI: 1.00–4.82) compared to those who had no education. Those who were married had reduced odds of 43% (adjusted odds ratio [AOR] = 0.57, 95% CI: 0.36–0.92), those who were widowed had reduced odds of 52% (AOR = 0.48, 95% CI: 0.21–1.07) and those who were cohabitating had reduced odds of 73% (AOR = 0.27, 95% CI: 0.08–0.91) of having a positive attitude compared to those who were single.

Secondary level of education, being divorced and being hypertensive were significantly associated with good practices. Adjusting for other variables, those who had secondary education had increased odds of 3.13 (95% CI: 1.29–7.61) of having good practices compared to those with no education. Those who were divorced had reduced odds of 66% (AOR = 0.34, 95% CI: 0.12–0.94) of having good practices compared to those who were single. Those who were hypertensive had increased odds of 2.46 (95% CI: 1.12–5.38) of having good practices compared to those who were not hypertensive, adjusting for other variables.

## Discussion

Of the participants in this study, 70% displayed good knowledge of COVID-19,^20,21^ and this knowledge translated into positive attitudes and practices towards the disease. A few studies carried out respectively in Egypt, India and Iran showed similar findings.^3,6,13,22^

Contrary to our findings, some studies in Africa found that a significant number of chronic disease patients had poor knowledge of and practices towards COVID-19.^2,23^ This difference in knowledge is perhaps because after the fourth wave of COVID-19, at the time of this study in South Africa, most of the population had more information on COVID-19 than in other countries.

This study found a significant association between knowledge about COVID-19 and the younger age group. Chronic patients aged 70 years and above had poor knowledge of the disease, with poor attitude and practices. It has been established that older patients, particularly those older than 65 years, or patients with comorbidities, had a high rate of admission to hospital (including the intensive care unit) and a high mortality rate.^24,25,26,27,28,29,30^ The poor KAP observed in this age range is of concern. Supporting this category of patients will alter the admission and mortality rate. The severe acute respiratory syndrome coronavirus 2 (SARS CoV-2) vaccine is the most recommended preventative measure.

In this study, level of education was associated with good knowledge of and good practices towards COVID-19. As expected, having a tertiary level of education correlated with better knowledge. These findings are consistent with those observed elsewhere.^6,31,32^

This study found that non-hypertensive and HIV-positive patients had a good knowledge of COVID-19 compared to hypertensive patients and those who did not have HIV. This knowledge translated into a more positive attitude towards COVID-19 among HIV-positive patients than in HIV-negative patients. This may be due to the fact that patients with comorbidities perceived themselves as at risk of COVID-19, and not only seek knowledge but improve their attitudes and practices to protect themselves.

Hypertension was most associated with COVID-19 compared to other chronic conditions. The attitude displayed by hypertensive patients is crucial in combatting COVID-19,^6^ as the risk associated with COVID-19 in hypertensive patients has been widely described.^29,33^

Human immunodeficiency virus was the second most common chronic condition associated with COVID-19, and the KAP towards the disease among HIV patients was commendable. This finding is consistent with those of other studies.^34^

The study’s strength lies in its emphasis on the need for patients aged 70 and above, who may require special attention due to their poor KAPs towards COVID-19.

Although it is the largest in the North West province, this study depicts just one health district. Additionally, the cross-sectional nature of the study did not allow us to show the cause–effect relationship. Moreover, because the KAP was self-reported, this may not be what is actually the case. For these reasons, further study is warranted.

The study found that the level of knowledge about COVID-19 was generally good among the participants, which translated into a positive attitude and practices towards the disease. However, older patients had poorer knowledge of and practices towards the disease, which could lead to higher hospitalisation and mortality rates. A tertiary level of education was associated with better knowledge of and practices towards COVID-19.

The study suggests that improving knowledge of and attitudes towards COVID-19, particularly among older patients and those with comorbidities, could be beneficial in reducing hospitalisation and mortality rates.
